# The Emerging Role of Branched-Chain Amino Acids in Liver Diseases

**DOI:** 10.3390/biomedicines10061444

**Published:** 2022-06-18

**Authors:** Emily Kwun Kwan Lo, Felicianna, Jing-Hang Xu, Qiao Zhan, Zheng Zeng, Hani El-Nezami

**Affiliations:** 1 School of Biological Sciences, University of Hong Kong, Pokfulam, Hong Kong 999077, China; emilylkk@hku.hk (E.K.K.L.); feli19@hku.hk (F.); 2 Department of Infectious Diseases, Peking University First Hospital, Peking University, Beijing 100034, China; ddcatjh@sina.com (J.-H.X.); dralettazhan@bjmu.edu.cn (Q.Z.); 3 Institute of Public Health and Clinical Nutrition, School of Medicine, University of Eastern Finland, FI-70211 Kuopio, Finland

**Keywords:** branched-chain amino acids, liver diseases, non-alcoholic fatty liver disease, cirrhosis, hepatocellular carcinoma

## Abstract

Chronic liver diseases pose a substantial health burden worldwide, with approximately two million deaths each year. Branched-chain amino acids (BCAAs)—valine, leucine, and isoleucine—are a group of essential amino acids that are essential for human health. Despite the necessity of a dietary intake of BCAA, emerging data indicate the undeniable correlation between elevated circulating BCAA levels and chronic liver diseases, including non-alcoholic fatty liver diseases (NAFLD), cirrhosis, and hepatocellular carcinoma (HCC). Moreover, circulatory BCAAs were positively associated with a higher cholesterol level, liver fat content, and insulin resistance (IR). However, BCAA supplementation was found to provide positive outcomes in cirrhosis and HCC patients. This review will attempt to address the contradictory claims found in the literature, with a special focus on BCAAs’ distribution, key signaling pathways, and the modulation of gut microbiota. This should provide a better understanding of BCAAs’ possible contribution to liver health.

## 1. Introduction

In recent years, chronic liver diseases (CLDs) have become more common worldwide. It is estimated that over eight million people are currently suffering from CLDs [1]. CLDs are characterized by progression from chronic hepatitis, fibrosis, and cirrhosis to hepatocellular carcinoma (HCC). In 2017 alone, over two million people died from liver-related deaths worldwide [2]. Liver diseases are, therefore, a major global health-related burden. Non-alcoholic fatty liver disease (NAFLD) is a major public health issue due to its high and rising global prevalence rate. NAFLD and HCC share similar risk factors, including obesity, type 2 diabetes (T2D), and metabolic disorders [3,4]. The manifestation of HCC is, therefore, common in patients with chronic liver diseases, including alcoholic liver disease, non-alcoholic steatohepatitis, chronic hepatitis, and liver cirrhosis [5,6,7].

Amino acids (AA) are an essential nutrient for human health. The building block of proteins are amino acids, which are separated into two main categories: essential amino acids and non-essential amino acids. Essential amino acids are amino acids that cannot be synthesized by humans, and thus have to be supplied from an exogenous diet, while non-essential amino acids are amino acids that can be synthesized in the body [8]. One group of essential amino acids is branched-chain amino acids (BCAAs: leucine, valine, isoleucine), which contain aliphatic branched side chains. BCAAs not only provide an essential substrate for protein synthesis, but also contribute to energy homeostasis, including gluconeogenesis and lipid metabolism [9]. Alterations in plasma AA levels were found in patients with obesity, diabetes, and type 2 diabetes [10,11,12], and recently studied metabolomic reports showed an elevated circulatory BCAAs level in multiple liver diseases [10,13,14]. Indeed, a low serum Fischer’s ratio (BCAAs to aromatic AA ratio) has been defined as a hallmark of liver cirrhosis [13]. Moreover, increasing circulatory levels of BCAAs were found to be associated with both an increase in triglyceride levels and a decrease in high-density lipoprotein (HDL) cholesterol level [14]. However, studies have revealed the promising effect of BCAA supplementation on ameliorating liver diseases [15,16,17]. This review is an attempt to explore the contradictory role of BCAAs in liver diseases and provide insights regarding new findings on the contribution and protective effect of BCAAs and their mechanisms on liver diseases. More recently, gut microbiota were reported to play a crucial role in modulating the bioavailability of BCAAs through regulating BCAA transporters [18]; accordingly, the contribution of gut microbiota to BCAAs’ role in liver diseases will be discussed.

## 2. Circulation of BCAAs

The processes of BCAAs’ synthesis and metabolism have been extensively reviewed [19,20]. In this section, an overview of BCAAs’ enterohepatic circulation and signaling pathways will be presented to support a later discussion on the role of BCAAs in liver inflammation and carcinogenesis.

### 2.1. BCAA Transport and Metabolism

**Figure 1:**
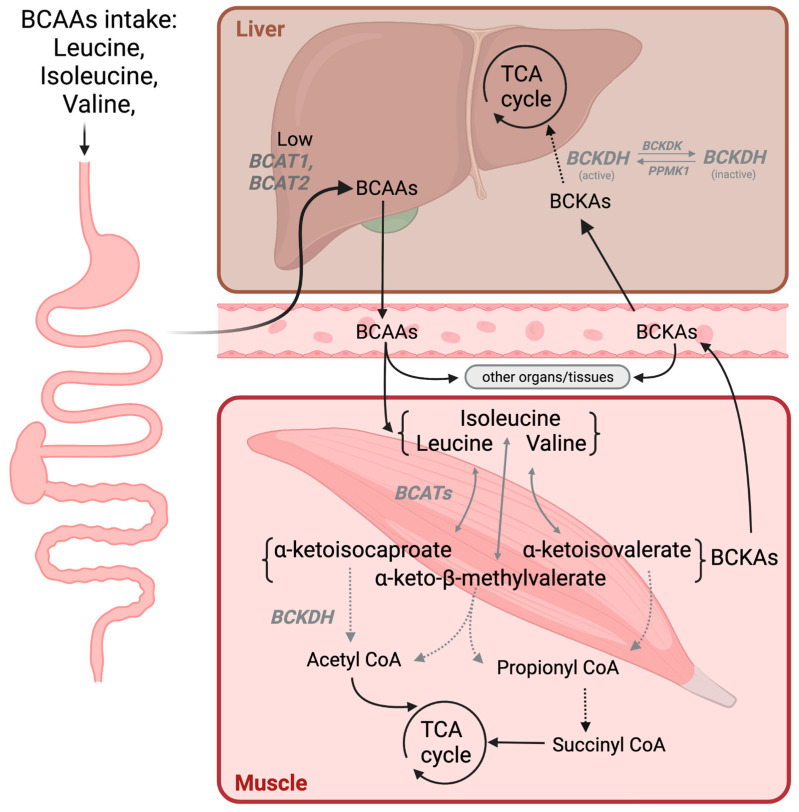
Schematic representation of BCAAs’ metabolism. Solid arrow represents the single metabolic steps, and dotted arrows represent simplified multistep processes. BCAAs, branched-chain amino acids; BCATs, branched-chain amino transferases; BCKDC, branched-chain alpha-keto acid dehydrogenase. Figure created with BioRender.com, accessed on 13 April 2022 (San Francisco, CA, USA).

It is known that, upon oral intake of BCAAs, they circulate in the bloodstream, by-passing the first-pass metabolism in the liver due to the low activity of branched-chain amino transferases [21]. Branched-chain amino transferases BCAT1 and BCAT2 are the first enzymes to degrade BCAAs. They catalyze the reversible conversion of BCAAs to branched-chain α-ketoacids (BCKAs)—leucine to α-ketoisocaproate, valine to α-ketoisovalerate, and isoleucine to α-keto-β-methylvalerate—by transferring the amino groups to α-ketoglutarate (Figure 1). BCATs are found in many tissues, but are mostly expressed in the skeletal muscle; thus, they form the main metabolism site of BCAAs, with over half of the total circulating BCAAs ending up there, while a quarter enter the splanchnic circulation, and the remainder are used up by the brain and other tissues [22]. Once converted into BCKAs, they can undergo a series of irreversible enzymatic reactions, or move into the circulation for decomposition in other tissues.

It is important to note that muscle tissues are not gluconeogenic; thus, if these BCAAs (i.e., valine or isoleucine) cannot be fully utilized by the muscles, they must be removed (either in their original form or their metabolic products). The transformation of BCKAs by branched-chain α-keto acid dehydrogenase (BCKDH) is the next step in BCAA catabolism, and this enzyme is also known to be the rate-limiting step in the BCAAs’ catabolism pathway. Notably, its levels are very low in skeletal muscles, and are the highest in the liver and the heart [23]. Thus, upon conversion into BCKAs and their release into the circulation from the skeletal muscle, the liver mainly extracts and decomposes them. Indeed, the muscles are known to play an important role in producing gluconeogenic substrates from BCAAs for the liver [24]. For instance, 3-hydroxyisobutyrate (3-HIB), a metabolite of valine, is well-known to act as a gluconeogenic substrate in the hepatocytes [9,23].

### 2.2. BCAAs’ Signaling and Its Benefits

**Figure 2:**
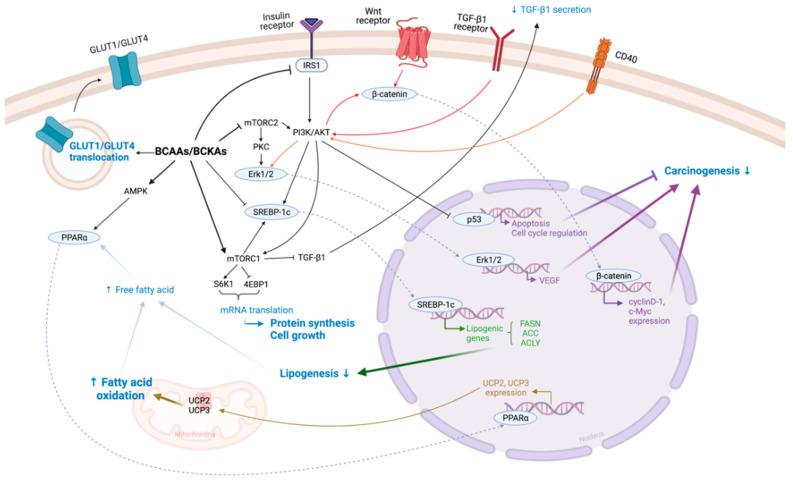
Schematic representation of BCAAs’ signaling. Solid arrow represents the single metabolic steps, and dotted arrows represent its translocation into the nucleus. BCAAs, branched-chain amino acids; BCKAs, branched-chain α-keto acids; GLUT, glucose transporter; IRS1, insulin receptor substrate; PI3K/AkT, phosphatidylinositol 3-kinase/protein kinase B; mTORC, mTOR complex; S6K1, serine/threonine protein kinase; 4EBP1, eukaryotic initiation factor 4E-binding protein 1; SREBP-1c, sterol regulatory binding protein 1c; PKC, protein kinase C; Erk, extracellular signal-regulated kinase; TGF-β1, transforming growth factor beta 1; AMPK, AMP-activated protein kinase; VEGF, vascular endothelial growth factor; PPARα, peroxisome proliferator-activated receptor α; UCP, uncoupling proteins; FASN, fatty acid synthase; ACC, acetyl-CoA carboxylase; ACLY, ATP citrate lyase. Figure created with BioRender.com, accessed on 13 April 2022 (San Francisco, CA, USA).

BCAAs can trigger different types of signaling (Figure 2), depending on the condition of the host’s body, i.e., energy homeostasis. Firstly, BCAA consumption increases the amino acid pool and plasma insulin levels. In cases of severe energy depletion, such as during endurance training or starvation, in addition to an increase in insulin caused by BCAAs, AMP-activated protein kinase (AMPK) is activated, which in turn redirects nutrients, including BCAAs that are consumed to undergo gluconeogenesis and form products to be oxidized for ATP generation [23]. An increase in insulin also enhances the translocation of glucose transporters GLUT1 and GLUT4 in intestinal and muscle cells to increase glucose uptake for ATP production [25].

On the other hand, BCAA consumption during rest or exercise recovery triggers a pathway that redirects the BCAAs’ metabolism into protein synthesis or restoring and building up glycogen storage in muscles/liver. The increase in insulin triggers the activation of insulin receptor substrate (IRS1) and leads to the activation of the phosphatidylinositol 3-kinase (PI3K)/protein kinase B (AKT) signaling pathway, which in turn activates mTOR complex 1 (mTORC1) via the phosphorylation of TSC1/2, while the increase in the amino acid pool directly triggers the mTORC1 pathway. This pathway plays the important role of maintaining cell proliferation, cell cycle, angiogenesis, apoptosis, and metabolism [26], and is best elaborated in muscle cells to trigger protein synthesis and repress protein degradation. Thus, BCAAs are a popular supplement for athletes, which support muscle growth and maintenance [27]. A downstream activator of mTORC1 includes serine/threonine protein kinase (S6K1) and eukaryotic initiation factor 4E-binding protein 1 (4EBP1), which are greatly enhanced by BCAAs, but particularly leucine [28]. S6K1 and 4EBP1 are both known to be involved in the regulation of mRNA translation [29]. The activation of S6K1 is also known to bring about a negative feedback loop in the activation of insulin signaling, which can suppress the activity of IRS1, and thus inhibit the downstream signaling of PI3K/Akt. However, the suppression of IRS1/PI3K/Akt signaling is rarely observed in muscle cells, especially during exercise recovery [30,31].

BCAAs and their metabolites were found to be able to attenuate PI3K/Akt signaling on other tissues, such as the liver, and this was thought to be the main mechanism by which BCAAs bring about beneficial health outcomes beyond nutrition [29,32]. The attenuation of the PI3K/Akt pathway is beneficial because this pathway is known to be involved in cell survival pathways, glucose homeostasis, and lipid synthesis [33]. It is known that Akt activation mediates the suppression of p53, a well-known tumor-suppressor protein involved in the apoptosis of cancer cells [34]. Additionally, it has recently been shown that both mTORC1 and Akt activation are required for the activation of the transcription factor sterol regulatory element-binding protein 1c (SREBP-1c), which is known to induce the transcription of lipogenic genes, such as fatty acid synthase (FASN), acetyl-coA carboxylase (ACC), and ATP citrate lyase (ACLY) [35]. Hence, the attenuating effect that BCAAs have on the PI3K/Akt pathway could extend to the expression of SREBP-1c and its downstream effectors in the liver [36,37].

In a hepatic tumor cell model, BCAA supplementation has been found to inhibit cell proliferation via decreasing the activity of the PI3K/Akt pathway [38]. Furthermore, BCKAs were also found to be able to downregulate the mTOR complex 2 (mTORC2) pathway, with downstream signaling effectors including Akt and Protein Kinase C (PKC) [39]. The dysregulation of PKC and PI3K/Akt signaling has been observed to be associated with many and all human cancers, respectively, and the latter is known to be the main contributor to tumor development and progression [40]. For instance, activated Akt was found to be the mediator in CD40-induced vascular endothelial growth factor (VEGF) production, a well-known protein that is upregulated in tumor cells [41]. Meanwhile, downstream signaling of PKC involves the direct activation of the RAF/MEK/Erk signaling pathway, in which the hyperactivation of this pathway is also associated with many human cancers [42]. Additionally, through the activation of mTORC1, BCAAs were found to reduce the expression of transforming growth factor beta 1 (TGF-β1) cytokines in both hepatic stellate cells and mouse hepatocytes [43]. Hepatic stellate cells are involved in the formation of fibrosis: its activation is brought on during liver injury and they proliferate, contract, and perform chemotaxis across the liver [44]. Activated stellate cells will secrete TGF-β1 and collagen to form of scar tissue and, if they remain activated, result in cirrhosis of the liver. Particularly, the authors reported that TGF-β1-induced Wnt/β-catenin signaling and pro-apoptotic signaling were also suppressed [43]. Overexpression of the β-catenin signaling pathway is known to be involved in carcinogenesis, including hepatocellular carcinoma (HCC), as it is found to promote the expression of oncogenes including cyclinD-1 and c-Myc [45]. Furthermore, BCAAs elevate peroxisome proliferator-activated receptor α (PPARα) and its downstream expression of uncoupling proteins 2 (UCP2) and UCP3 in the liver and muscle, respectively, which leads to the increased oxidation of free fatty acids [46]. These findings imply that BCAA consumption may help with the progression of liver diseases, particularly cancers.

## 3. Circulatory BCAAs Level as an Indicator of a Dysmetabolic State

### 3.1. High Circulatory BCAAs Level in NAFLD Patients

In contrast to the documented beneficial effect of BCAA supplementation in cell culture models, higher BCAA circulatory levels were found in NAFLD patients [47,48,49]. The rise in BCAA levels has also been positively associated with insulin resistance (IR) and total cholesterol and glycerol levels in type 2 diabetes (T2D) and obese patients [50,51]. Since T2D and obesity are known to be risk factors for non-alcoholic fatty liver disease (NAFLD) and non-alcoholic steatohepatitis (NASH) [52], this raised the question of whether the BCAA level is influenced by these underlying risk factors.

A large-scale clinical study on NAFLD subjects without T2D provided insight into the synergistic effect of NAFLD and the elevated BCAA levels on the development of type 2 diabetes. The total plasma BCAAs were positively correlated with a high fatty liver index (FLI), which was calculated from the levels of blood triglycerides, blood gamma-glutamyl-transferase, BMI, and waist circumference. In the 7.3-year follow-up analysis, nearly 20% of patients with elevated FLI were found to develop T2D. This elevation was suggested to be linked with the impaired hepatic mitochondrial function and increased mitochondrial lipid β-oxidation in NAFLD [47]. Although there is no causative relationship between T2D and the high BCAA circulatory levels in NAFLD, BCAA levels were positively correlated with T2D incidence [53]. The activity and expression of BCAA catabolic enzymes were previously reported to be altered in pathologic conditions involving metabolic disorders, in which they are downregulated in patients with type 2 diabetes [54].

Patients with both NAFLD and obesity have a higher BCAA circulatory level than non-obese NAFLD patients. In an Italian cohort with non-obese NAFLD patients, obese NAFLD patients, and healthy subjects, the rise in BCAA levels was more profound in the obese NAFLD group when compared to the healthy control, while valine and isoleucine levels were only significantly higher in the obese NAFLD group [55]. These findings aligned with observations from a study on NAFLD patients with severe obesity, where plasma BCAAs were positively correlated with steatosis stages and liver fat content [10]. A recent study illustrated the metabolic differences between obese subjects with and without progression to NAFLD. BCAAs were found to be increased in NAFLD-obese patients, but not in obese or lean healthy subjects. Further univariate analysis identified isoleucine as one of the factors that discriminates between obese patients vs. obese NAFLD patients. This study highlighted the crucial association between impaired BCAA metabolism and the manifestation of NAFLD [56]. In obese NAFLD patients, a higher consumption of BCAAs was associated with worse hepatic health in terms of liver fat content [57].

The elevated BCAAs levels were also found to contribute to IR. IR was also found to be positively correlated with the rise in BCAA levels in NAFLD and fibrosis patients [47,58]. The circulatory levels of BCAAs were positively correlated with the insulin-resistance index, HOMA-IR [58]. It was suggested that BCAAs may lead to IR through activating the mTORC1 signaling pathway, which produces the chronic phosphorylation of mTOR and IRS1_Ser307_ [9]. However, recent findings found that an increase in mTORC1 signaling from BCAA consumption alone would not affect insulin sensitivity in the long term [59].

The rise in plasma BCAA levels in NAFLD patients was also found to be sex-dependent. Male subjects were found to have significantly higher BCAA levels than female subjects. Plasma BCAA levels in female subjects were correlated with NAFLD and fibrosis stages, while the opposite result was found in male subjects. Leucine and valine were inversely correlated with NAFLD stages in males. Nevertheless, without considering the gender differences, leucine and isoleucine were significantly associated with NAFLD stages [60].

Since circulatory BCAA levels were consistently found to be significantly increased in liver diseases, the possibility of using circulating BCAAs’ concentration as a diagnostic tool was suggested. A study on obese children found a high area under the curve (AUC), 0.92 (95% confidence interval 0.83–1.00), for using BCAA to discriminate between severe steatosis and a healthy obese subject, while an AUC of 0.82 (95% CI 0.67–0.97) could be used for the discrimination of any steatosis [10]. The elevation in BCAAs was not limited to their systematic levels. BCAA level was elevated in liver tissue in NASH patients vs. healthy subjects. However, the liver BCAA levels were found to be unchanged in simple steatosis/NAFLD patients vs. healthy subjects [61]. Although the study only included data from a limited number of patients, it suggested that the change in systematic levels is aligned with the local level in NASH patients, which might contribute to the activation of the aforementioned mTOR pathway [62].

### 3.2. Rising BCAA Levels in HCC Patients

Plasma BCAA levels were found to be significantly increased and have been identified as a biomarker of progression to HCC [63]. A low BCAAs/tyrosine ratio (≤4.4) was found to be a prognostic factor for HCC patients with chronic liver diseases. The BCAAs/tyrosine ratio was significantly negatively correlated with the liver function marker, albumin albumin-bilirubin (ALBI) [64,65].

The rise in BCAAs was not limited to their systemic level. A recent study found an increase in tissue BCAA level in HCC patients with severe fibrosis and cirrhosis. In 52 paired HCC tumor and nontumor tissues, BCAAs were found to be elevated in HCC tissue when compared with adjacent non-tumoral tissues [66]. The same finding was also found in another study with paired HCC tumor and nontumor tissues from 48 of their patients [67]. The team took a further look into the transcriptomic profile of HCC tumors and adjacent tissues of patients in both Singapore General Hospital and data from the Cancer Genome Atlas [67]. They found that the BCAA degradation pathway was a significantly enriched KEGG pathway in the tumors of both their 48 HCC patients and the HCC cohort from the Cancer Genome Atlas. More than 40 BCAA catabolic enzymes, including BCKDH and acyl-CoA dehydrogenase enzymes (ACADs), were suppressed in tumors. The accumulation of BCAA in the tumor activated mTORC1 signaling. A higher expression of the catabolic enzyme of BCAA was, therefore, linked to better survivability for patients. The group further investigated the impact of BCAAs on tumor development by using diethylnitrosamine (DEN)-injected high-fat diet-fed mice. Tumor number and size were elevated in the BCAA-fed group. Consistent with their findings in human subjects, BCAA catabolic enzymes were suppressed in BCAAs/DEN-injected mice, while they were enhanced in control mice fed with BCAAs.

In livers of HCC patients, and animal models, including high-fat diet-induced obesity and HCC tumor models, BCKDH activity and expression were found to be downregulated, and BCKDH kinase (BCKDK), the enzyme responsible for suppressing the activity of BCKDH, was found to be upregulated [23]. The consequence of this is an inability to fully oxidize BCKAs. The accumulation of BCKAs, especially from valine and isoleucine metabolism, may lead to mitochondrial dysfunction. It was previously reported that increased BCKA levels suppress the expression of succinate dehydrogenase, which affects the TCA cycle and the electron transport chain [68]. As a result, acylcarnitine byproducts were formed instead of the complete TCA cycle, and this elevation of plasma acylcarnitine is considered a marker of IR, type 2 diabetes, and cardiovascular diseases [23]. Meanwhile, in an animal and human HCC tumor model, the dysregulation of BCAA oxidation was found to induce chronic mTORC1 activation [67].

## 4. BCAA as a Treatment for Liver Diseases

### 4.1. BCAA as a Therapeutic Treatment in Humans

Despite the association between elevated blood BCAA levels and negative conditions in liver diseases, the consumption of BCAA supplements was previously linked to a beneficial outcome in various liver diseases, especially during advanced fibrosis or cirrhosis, and especially hepatic encephalopathy. BCAA supplementation is recommended to cirrhotic patients according to the guidelines of the American Association for the Study of Liver Diseases (AASLD) and the European Association for the Study of the Liver (EASL) [69]. Table 1 summarizes the ongoing clinical trials utilizing BCAAs to treat liver diseases.

In three separate studies, the supplementation of BCAAs in the diet of patients with advanced liver cirrhosis resulted in a significant improvement in major cirrhosis-related events, including improvements in Child–Pugh (CP) score, MELD score, and/or a significantly higher number of patients with event-free survival [70,71,72]. The beneficial effect of BCAA supplementation was not limited to cirrhotic patients. BCAA supplementation was also found to be useful in preventing the occurrence of HCC in cirrhotic patients [73]. The majority of HCC patients (80–90%) were diagnosed with underlying cirrhotic conditions [74]. Although there have been few human trials on BCAA supplementation in HCC patients, increasing evidence from animal studies provides an indication of the potential beneficial effect of BCAAs.

**Table 1:** Clinical trials and ongoing clinical studies utilizing BCAA to treat liver disease.

Type of Studies *	Interventions	Patients/Control	Sample Size	Duration	Outcome #/Outcome Measures *	Ref.
Multicenter RCT	VAL, LEU, ILE	Advanced liver cirrhosis	232	6 months	- MELD, CP score, - Cumulative cirrhosis-related event-free survival	[70]
Double-blinded RCT	VAL, LEU, ILE	Advanced cirrhosis	174	12 months	- CP score - Total bilirubin level - Death or deterioration of symptoms	[71]
N/A	VAL, LEU, ILE /AAA	Cirrhosis	104	>6 months	- Cumulative survival rate - Delayed complication including hepatic failure and gastrointestinal bleeding	[13]
N/A	VAL, LEU, ILE	Cirrhosis	211	≥6 months	- HCC occurrence - Event-free survival rate	[73]
Single-blinded RCT	AXA1665 (Leu: Ile: Val)	Child–Pugh A and B Cirrhosis	16	15 days	- Liver Frailty Index - Leaner body composition	[75]
Single-blinded, Multicenter RCT	AXA1125 (VAL, LEU, ILE, ARG, GLN)	Patients with NAFLD with and without T2D	102	16 weeks	- ALT, K18 - Fibro-inflammation marker, cT1, Pro-C3	[16]
RCT	VAL, LEU, ILE	HCC	51	12 months	- Intrahepatic recurrence rate - Event-free survival	[72]
Ongoing clinical studies						
Triple-blinded RCT, Phase II	AXA1125 (VA, LEU, ILE, ARG, GLN)	NASH with fibrosis	273	48 weeks	Improvement in steatohepatitis, resolution of NASH/ fibrosis	[76]
RCT	VAL, LEU, ILE	Cirrhosis	60	3 months	Muscle mass, insulin-resistant	[77]

* Primary outcome measures and secondary outcomes that related to liver health for ongoing clinical trials. VAL, valine; LEU, leucine; ILE, isoleucine; ARG, arginine; GLN, glutamine; SER, serine; CP, Child–Pugh score; MELD, model for end-stage liver disease; NASH, non-alcoholic steatohepatitis; RCT, randomized clinical trials; ALT, alanine aminotransferase; K-18, keratin 18.

### 4.2. BCAA as a Prophylactic Treatment of Liver Diseases in Animals

A DEN-injected rat liver injury model showed that BCAAs significantly lowered dysplastic nodules. Although BCAAs could not prevent progression to malignant tumors, the supplementation prevented liver neoplasm lesions [17]. This effect was due to the suppression of tumor angiogenesis as a result of the low secretion of VEGF. BCAA(s) was also previously found to boost the efficacy of the chemotherapy drug, cisplatin, which is widely used for the treatment of cancers. The supplementation of leucine increased cisplatin sensitivity by activating the mTOR pathway [78].

Over the course of NAFLD/NASH progression, cirrhosis may also develop; therefore, BCAA supplementation has also been increasingly investigated to treat these diseases, and/or prevent them from progressing to cirrhosis. Although there is a lack of human studies utilizing BCAAs to treat NAFLD or NASH patients, some animal studies have pointed to a potential positive outcome of its utilization, although the results are controversial and not conclusive. In a choline-deficient, high-fat diet-induced NASH mice model, BCAA lowered serum ALT levels and hepatic triglyceride, while the liver histology showed that the lipid droplet area and fatty acid synthase (FAS) were lowered [79]. Similar results were obtained from high-fat (45%) diet NAFLD rat and obese mice models, where BCAA supplementation decreased fat accumulation and triglyceride concentration in the liver, and significantly lowered the steatosis score [46,80]. However, several studies highlighted that while BCAA supplementation reduced hepatic triglycerides, body weight, and food intake, hepatic IR could not be improved and a persistent induction of mTORC1 activation was observed, implying that the supplementation of BCAAs worsens the underlying metabolic disorder [15,37,81]. The persistent mTOR activation arose from the combination of both high-fat and BCAA supplementation, and this IR could be reversed using the mTORC1 inhibitor, rapamycin [9]. In contrast, rapamycin could not reverse high-fat diet-induced IR. Furthermore, BCAA-supplemented normal chow-feeding in rats did not induce increased mTOR activation [9]. This indicates that BCAA-high-fat-induced IR is likely to be more reversible compared to only high-fat-induced IR upon adopting a healthier diet. Furthermore, a previous survey conducted on the typical human Western diet found that the diet only contains around ~35% fat; hence, a review article suggested the use of diets with ~45% fat in rodents to confer a better rodent and human inter-study agreement [37]. Contradictory observations were found in the studies by Muyyarikkandy et al. and Zhao et al., who adopted a 60% fat rodent diet; thus, these observations may not necessarily be duplicated in humans. Indeed, a clinical trial of 102 NAFLD patients found that BCAA supplementation significantly lowered both liver disease markers (i.e., ALT and keratin-18 (K18)) and fibrosis markers [16]. With this, and the abundant evidence that BCAAs could help in liver cirrhosis, it should not be of great concern that BCAAs may exacerbate the disease condition if a healthy balanced diet is adopted during the intervention. On the other hand, the overall impression of these studies highlights the complex relationship between diet, BCAA, liver health, and IR, while also bringing attention to the gut–liver axis.

The full mechanism of how BCAAs prevent further deterioration in chronic liver diseases remains largely unclear. An explanation for this may be that the supplementation of BCAAs could elevate its catabolism via directly affecting the levels of its catabolizing enzyme. In particular, the increase in PPAR-α expression by BCAAs, through AMPK and an increase in serum-free fatty acid levels, could prevent the increase in BCKDK activity, preventing the suppression of BCKDH activity in catabolizing BCKAs [82,83]. The contribution of PPAR-α to lipid homeostasis was found to be crucial to preventing steatosis-induced NASH development [84]. It is also important to note that the loss of muscle mass, the major BCAA catabolic site, is usually accompanied by chronic liver diseases [85,86]. Improving the muscle mass [87] could potentially benefit muscle BCAA catabolism and its subsequent glutamine synthesis. The increase in plasma glutamine (GLN) was observed via the supplementation of BCAAs, along with a lowering of plasma glutamate (GLU) [88]. This increase in GLN availability was found to be beneficial to the immune system and the production of the natural antioxidant glutathione, which is beneficial to liver health [89,90].

## 5. BCAA Promotes Hepatic Health through Modulation of Gut Microbiota

In recent decades, mounting evidence has unveiled the crucial role of gut microbiota in metabolism. Our gut is home to a large amount of gut microbiota, from fungi and archaea to bacteria. Recent evidence has confirmed the crucial contribution of gut microbial dysbiosis to NAFLD pathogenesis, scrutinizing the importance of homeostasis in the gut–liver axis. Shotgun sequencing results from feces of metabolic-associated NAFLD patients displayed an elevated abundance of ethanol-producing bacteria and a decreased abundance of butyrate-producing bacteria [91,92]. In obese NAFLD patients, steatosis was positively correlated with dysregulation of the microbial BCAA metabolism, in which its biosynthesis is upregulated [15]. Therefore, the increase in plasma BCAAs observed with obesity and many insulin-resistance-associated diseases, including NAFLD, is likely due to the dysbiosis in microbiota instead of oral consumption. Indeed, with most studies showing beneficial effects on liver health, it is thought that the consumption of BCAAs may alter the gut microbiota composition and consequently reduce the circulatory BCAA level.

Several rodent studies have provided evidence on how gut microbiota regulate BCAA levels and subsequently contribute to liver disease. A study on BCAA supplementation to rats fed a high-fat diet showed that BCAAs increased the beneficial gut microbiota *Ruminococcus flavefaciens*. Iwao et al. showed that cellulose was necessary for the beneficial effect of BCAAs, which highlighted the involvement of gut microbiota [80]. BCAA supplementation was also found to lower the abundance of Proteobacteria, a phylum that includes pathogenic bacteria, while increasing beneficial *Bifidobacterium* species in healthy mice [93]. The oral administration of *Bifidobacterium* strains to high-fat-fed mice showed an increase in GLP-1 secretion via the increase in short-chain fatty acids (SCFA) levels, particularly acetate [94]. The increase in GLP-1 secretion is likely to be beneficial to NAFLD patients, as it is known to increase insulin sensitivity and improve glucose metabolism [95].

A limited number of studies investigated the correlation between gut microbiota composition and circulating BCAA levels in patients with liver diseases. A recent study on healthy adolescence found fecal *Faecalibacterium prausnitzii* levels to be inversely correlated with serum BCAA levels, insulin levels, and HOMA-IR. *Faecalibacterium prausnitzii* contributed to the majority of bacterial BCAA transporters’ gene count [96]. *Faecalibacterium prausnitzii* is one of the most common gut microbe species in healthy adults, accounting for more than 5% of the total population, and their abundance was decreased in steatosis patients [97,98]. The gavage of *F. prausnitzii* to mice fed a high-fat diet improves parameters related to hepatic health, including AST, ALT serum levels, improved glucose tolerance, and insulin sensitivity, and decreased steatosis in the liver [97].

In relation to BCAAs’ metabolism, other members of the gut microbiota, *Bacteroides vulgatus* and *Bacteroides dorei,* were reported to improve BCAAs’ catabolism in brown adipose tissue and improve systemic glucose tolerance and insulin sensitivity in high-fat diet-induced obesity mice [99]. These species were previously found to be downregulated in T2D individuals, and their high abundance in obese mice was found to confer protective effects, i.e., in preventing the mice from developing T2D and NAFLD [100]. However, contrary to the findings by Yoshida et al., a separate study conducted by Pedersen et al. found that an increase in *B. vulgatus* abundance is positively correlated with insulin resistance in NAFLD patients. The team found that in 277 non-diabetic insulin-resistance patients, *Prevotella copri* and *Bacteroides vulgatus* were identified as the main species promoting insulin resistance by driving bacterial BCAAs’ synthesis [18]. Similarly, a separate study in 86 NAFLD patients with or without advanced fibrosis also presented elevated *B. vulgatus* and *Eubacterium rectale* [101]. Thus, it is still unclear whether *B. vulgatus* also confer the same beneficial effects on improving BCAA catabolism if supplemented in NAFLD patients. Nonetheless, Pedersen et al. only reported the adverse effect of gavaging *P. copri,* in which glucose intolerance and serum BCAA levels were elevated in high-fat diet-fed mice. *Prevotella copri* enrichment was also found to be specifically enriched in advanced fibrosis in a study involving 39 NAFLD patients with fibrosis [102].

## 6. Conclusions

**Figure 3:**
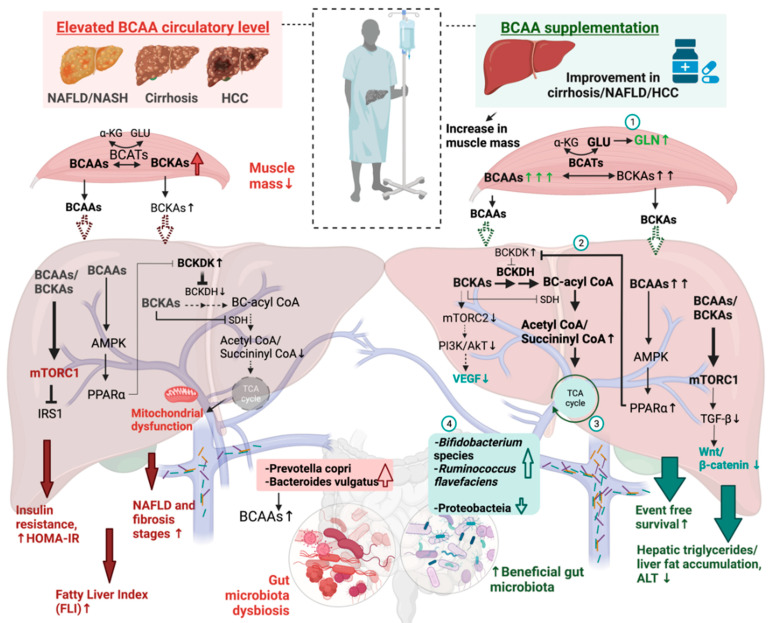
Schematic diagram depicting the conflicting role of BCAAs in liver diseases. The left (red) side represents a summary of known molecular pathways in the liver and gut microbiota dysbiosis upon elevated circulatory BCAAs that may contribute to the progression of liver diseases. The right (green) side summarizes the potential mechanisms that may explain how BCAA supplementation improves liver diseases’ outcomes. (**1**) Ingested BCAA undergoes transamination with α-ketoglutarate by BCATs, which generates glutamate, which is used in ammonia detoxification to glutamine. (**2**) Elevated BCAAs upregulate PPARα, which suppressed the rate-limiting enzyme of BCAAs’ catabolism, BCKDK. (**3**) Enhanced BC-acyl-CoA due to the lower suppression of BCKDH. (**4**) Supplementation increases beneficial gut microbiota while suppressing the phylum proteobacteria that includes pathogenic genera. BCAAs, branched-chain amino acids; AA, amino acid; BCATs, branched-chain amino transferases; BCKAs, branched-chain α-ketoacids; BCKDH, branched-chain alpha-keto acid dehydrogenase; BCKDK, BCKDH kinase; IRS-1, insulin receptor substrate 1; NAFLD, non-alcoholic fatty liver diseases; GLU, glutamate; GLN, glutamine; HCC, hepatocellular carcinoma; PI3K/AkT, phosphatidylinositol 3-kinase/protein kinase B; PPAR-α, peroxisome proliferator-activated receptor alpha; mTORC1, mTOR complex 1; BC-acyl CoAs, branched-chain acyl-CoAs; TGF-β1, transforming growth factor beta 1; VEGF, vascular endothelial growth factor. Figure created with BioRender.com, accessed on 13 April 2022 San Francisco, CA, USA).

In summary, recent studies proposed the possibility of utilizing BCAAs as a non-invasive marker for liver disease. While higher circulatory levels of BCAAs were found in NAFLD, NASH, cirrhosis, and HCC patients, the supplementation of BCAAs was found to be beneficial in liver diseases. The contradictory role of BCAAs could be due to the varied gut microbiota composition, in which the supplementation of BCAAs increased beneficial gut microbiota: *Ruminococcus flavefaciens* and/or *Bifidobacterium* species, vs. the contrasting involvement of the gut microbiota *Bacteroides vulgatus* and *Prevotella copri* in driving bacterial BCAAs’ synthesis in NAFLD patients (Figure 3).

## 7. Future Perspective

The current understanding and knowledge of the beneficial effects of BCAA supplementation in liver diseases remains inconclusive and is mainly derived from cell culture and animal studies, which cannot fully translate the etiology of human liver diseases and inter-individual variability. In terms of human studies, the majority were conducted without diet standardization, i.e., with a defined caloric intake and protein consumption. This is a crucial point to consider, since previous animal studies showed that diet is one of the crucial factors in chronic liver diseases, due to its relationship with the gut microbiome, especially in cases of NAFLD and NASH. Furthermore, limited research has been conducted to show how valine, leucine, or isoleucine, as compounds on their own, influence the outcome of liver diseases. Even though BCAAs share similar metabolic pathways and functions, it is unclear whether their beneficial effects rely on the combination of or an individual BCAA, as each BCAA has different metabolic effects. Additionally, prior research generally primarily focused on the clinical outcome of BCAAs’ administration, with few studies examining the correlation between BCAAs and gut microbiota in patients with liver disease and the mechanism of action. In recent years, the contribution of the gut–liver axis to the outlook of metabolic diseases has been extensively studied, but whether the gut microbiota could be the key regulator of the rise in BCAA levels is an area for future investigation.
